# Delineation of suitable sites for groundwater recharge based on groundwater potential with RS, GIS, and AHP approach for Mand catchment of Mahanadi Basin

**DOI:** 10.1038/s41598-023-36897-5

**Published:** 2023-06-18

**Authors:** Shreeya Baghel, M. P. Tripathi, Dhiraj Khalkho, Nadhir Al-Ansari, Aekesh Kumar, Ahmed Elbeltagi

**Affiliations:** 1 Department of Soil and Water Engineering, CTAE MPUAT, Udaipur, India; 2 Department of Soil and Water Engineering, SVCAET and RS, IGKV, Raipur, C.G. India; 3 Soil and Water Engineering, Department of Soil and Water Engineering, SVCAET and RS, IGKV, Raipur, C.G. India; 4 Department of Civil, Environmental and Natural Resources Engineering, Lulea University of Technology, 97187 Luleå, Sweden; 5 Department of Soil and Water Conservation Engineering, College of Technology, Govind Ballabh Pant University of Agriculture and Technology, Pantnagar, Uttarakhand 263145 India; 6 Agricultural Engineering Department, Faculty of Agriculture, Mansoura University, Mansoura, 35516 Egypt

**Keywords:** Environmental sciences, Hydrology

## Abstract

Groundwater management requires a systematic approach since it is crucial to the long-term viability of livelihoods and regional economies all over the world. There is insufficient groundwater management and difficulties in storage plans as a result of increased population, fast urbanisation, and climate change, as well as unpredictability in rainfall frequency and intensity. Groundwater exploration using remote sensing (RS) data and geographic information system (GIS) has become a breakthrough in groundwater research, assisting in the assessment, monitoring, and conservation of groundwater resources. The study region is the Mand catchment of the Mahanadi basin, covering 5332.07 km^2^ and is located between 21°42′15.525″N and 23°4′19.746″N latitude and 82°50′54.503″E and 83°36′1.295″E longitude in Chhattisgarh, India. The research comprises the generation of thematic maps, delineation of groundwater potential zones and the recommendation of structures for efficiently and successfully recharging groundwater utilising RS and GIS. Groundwater Potential Zones (GPZs) were identified with nine thematic layers using RS, GIS, and the Multi-Criteria Decision Analysis (MCDA) method. Satty's Analytic Hierarchy Process (AHP) was used to rank the nine parameters that were chosen. The generated GPZs map indicated regions with very low, low to medium, medium to high, and very high groundwater potential encompassing 962.44 km^2^, 2019.92 km^2^, 969.19 km^2^, and 1380.42 km^2^ of the study region, respectively. The GPZs map was found to be very accurate when compared with the groundwater fluctuation map, and it is used to manage groundwater resources in the Mand catchment. The runoff of the study area can be accommodated by the computing subsurface storage capacity, which will raise groundwater levels in the low and low to medium GPZs. According to the study results, various groundwater recharge structures such as farm ponds, check dams and percolation tanks were suggested in appropriate locations of the Mand catchment to boost groundwater conditions and meet the shortage of water resources in agriculture and domestic use. This study demonstrates that the integration of GIS can provide an efficient and effective platform for convergent analysis of various data sets for groundwater management and planning.

## Introduction

Groundwater is a critical natural resource for the efficient and cost-effective supply of industrial, agricultural, and domestic water in both urban and rural areas. As a result, it is crucial to human health as well as the health of several aquatic and terrestrial ecosystems. Around 36% of groundwater is extracted for residential consumption, 42% is utilised for agricultural production, and 27% is used for industrial uses globally^1^. More than 50% of urban residential water and 85% of rural water demands are currently satisfied by groundwater resources in India, with irrigation accounting for 92% of groundwater extraction^2^.

Since groundwater is a non-renewable natural resource that cannot be directly observed, it needs to be monitored and assessed properly to prevent overexploitation. Lack of effective water resource management leads to problems with water quality, salty water intrusion, water level drop, and other hydrogeological and geo environmental conflicts^3–5^.

Due to a substantial quantity of rainwater is lost through surface runoff, the problem of groundwater is worsening owing to an imbalance between groundwater recharge and exploitation^6,7^. If the essential actions are not implemented in a timely manner, India will face an acute water crisis in several of its states including the study present area and would be on the verge of a serious water catastrophe in the future^8^. As a result, the necessity for long-term management and development of the groundwater resource based on surplus surface runoff has arisen^9–11^. Identification of GPZs within the watershed is essential for accomplishing these goals. The GPZs can be identified in two ways: filed-based traditional approaches and modeling or soft computing techniques.

Numerous field-based classical methods have been employed to delineate GPZs using hydrogeological, geological and geophysical tools, however they are typically point-based, expensive, time-consuming, and lack of spatio-temporal information^12–18^. In recent years, several scientists have been working on enhanced methodological techniques for groundwater investigation, one of which is the frequency ratio^19,20^, logistic regression^17,18,21^, fuzzy logic^22,23^, Dempster-Shafer model^24,25^, weights of evidence model^26,27^, artificial neural network^28,29^, maximum entropy model^30^, and decision tree model^31,32^ have been successfully done.

Amongst the various MCDA approaches, the AHP is one of the most well-liked and scientific decision-making processes due to its structural simplicity, effectiveness, minimum bias, clarity, efficiency, and it also considers many watershed-influencing aspects^33–50^. AHP technique was developed by Thomas L. Saaty in the early 1980s^51^ for decision-making process. It offers various advantages and solves problems using both quantitative and qualitative methods^34,52,53^.

AHP integrated with geographic information system (GIS) provides the reliable spatio-temporal information of GPZs within the watershed^54^. Several researchers have reported on the use of GIS for delineation of GPZ to monitor and manage the groundwater resources^4,10,48,55–64^. As GIS helps to provides the spatial information and handle the big datasets. The weighted overlay analysis of the layer maps was prepared using the GIS, taking into consideration the priorities established by the AHP, and GPZs were identified^65–71^. The GPZs can further used for artificial groundwater recharge to improve groundwater storage by conserving the natural surface water flow. Artificial recharge techniques are commonly used to boost long-term productivity in regions where the aquifer has been depleted due to over-development^72,73^.

The present study area is a peri-urban catchment having extensive population growth, agriculture, and mining activities leads to increase the demand of water use. The study area also witnesses exponential growth in settlement, deforestation, and mining which increase the quick surface runoff rate and decrease the water retention time ultimately hinders in groundwater recharge. To meet the demand of water use, the extraction of groundwater has increased in the study area. The use of groundwater for agricultural purposes has led in a six-fold rise in groundwater draft in the study region during the previous 20 years^6^. Central groundwater board (2014) reported that the groundwater resources and demand fluctuates geographically and temporally due to the overexploitation of groundwater. Hence it become a paramount to identify the groundwater potential zones for management of groundwater resources by constructing the suitable groundwater recharge structures. Keeping view on the all aspect, in the present study AHP integrated with RS, GIS to reach the goals (1) to identify the groundwater potential zones, (2) to validate the groundwater fluctuation map, and (3) to provide appropriate sites for artificial recharge structures.

## Material and methodology

### Description of study area

**Figure 1:**
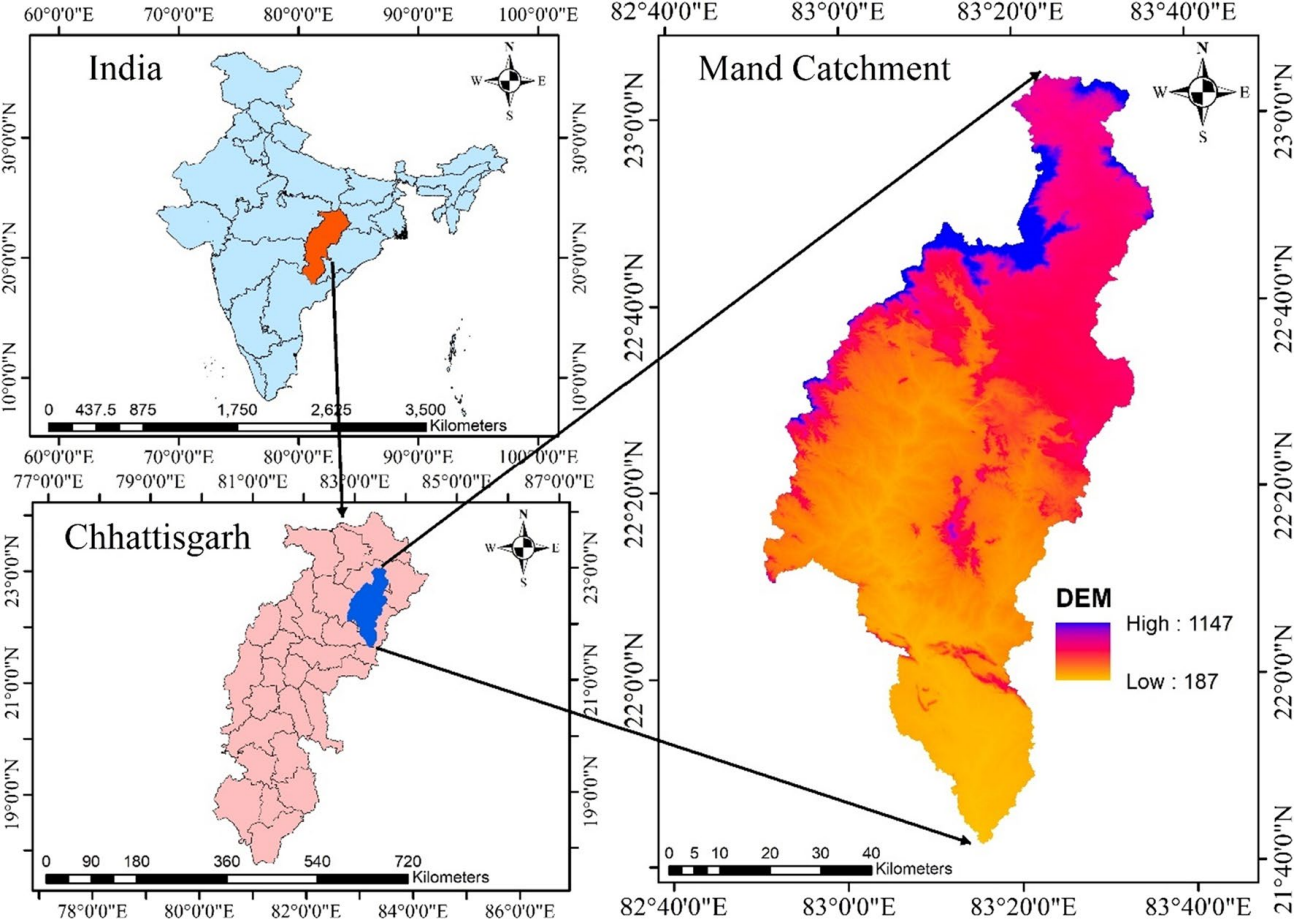
Study area map in Arc-GIS 10.4 (http://appsforms.esri.com/products/download/index.cfm?fuseaction=download.main&downloadid=1932).

The study area is the Mand river catchment of the Mahanadi basin, Chhattisgarh, India lies between the latitudes of 21°42′15.525″N and 23°4′19.746″N, and the longitudes of 82°50′54.503″E and 83°36′1.295″E (Fig. 1). The elevation of study area is ranges from 187 to 1147 m above mean sea level with the geographical area of 5332.07 km^2^. The Mand river originates in Surguja district's Bargidih village, and flows through Korba, Janjgir-Champa, Jashpur, and Raigarh district to the Mahanadi River at the eastern-part of Janjgir-Champa district. It runs north–south, then east–west, and then north–south and south-east. The major land use land cover along the river area is dense forest, agriculture, barren land (sand). In the agricultural crops mostly paddy, maize, and arhar are the main crops cultivated during *Kharif* season and wheat, gram, and linseed are cultivated during *Rabi* season.

A subtropical monsoon climate prevails in the Mand catchment, with three distinct seasons: summer, monsoon, and winter. In June, the southwest monsoon begins and lasts until mid-September and from October till February, the winter season lasts. From March until mid-June, the summer season is in full swing. The average annual precipitation of the study area was recorded as 1192.1 mm, where the southwest monsoon produces the most of it (85%), and it is the main source of groundwater recharge in the area. The average highest temperature was recorded in May is 42.5 °C, and the lowest temperaturewas recorded in January is 8.2 °C.

Geographically the Mand catchment is elongated, with high relief and steep to flat ground slope, causes high and quick surface runoff at the downstream river channel. The stream order in the watershed is first to fourth, indicating dendritic to sub-dendritic drainage and reflecting the steeply dipping rock strata. The higher the bifurcation ratio in the research region, the more structural disturbances there have been, and these structural disturbances have skewed the drainage pattern. The Ruggedness number (Rn), measure of surface unevenness value is high in the study region indicates that a terrain's structural complexity is extremely prone to erosion^74^.

### Data acquisition

Slope, geology, rainfall, drainage density, soil, geomorphology, lineament, land use land cover (LULC), and curvature were all analysed to establish a GPZs for the study area. The Digital Elevation Model (DEM) was obtained from the United States Geological Survey (USGS) (http://www.earthexplorer.usgs.gov) of the Shuttle Radar Topography Mission (SRTM) as 1 arc-second (approx. 30 m resolution). The DEM was used to delineate the catchment, sub-catchments boundary, slope, drainage and drainage density using various spatial analytic tools in ArcGIS software. The geology, geomorphology, lineament data, well location data, pre-monsoon and post-monsoon water level data were collected from Central Groundwater Board (CGWB) and runoff from Central Water Commission (CWC), (Bhubaneswar). The Inverse Distance Weightage (IDW) approach from ArcGIS 10.5 was used to generate the spatial distribution of rainfall map for the year of 2021^75^. Details of all the input data is mentioned in Table 1. The cloud-free sentinel-2 imagery was used to prepare the LULC map with supervised classification methods in ArcGIS 10.5.

**Table 1:** Different input parameters of the study.

Serial number	Description	Scale/data resolution	Year/range	Source
1.	Rainfall	0.25° × 0.25°	2021	https://chrsdata.eng.uci.edu
2.	SOI toposheet	1:50,000	1960	Survey of India, Raipur
3.	Sentinal-2	10 m	November 2021	USGS Earth Explorer http://www.earthexplorer.usgs.gov
	SRTM-DEM	30 m	2021	http://www.earthexplorer.usgs.gov
4.	Soil map	1:250,000	1976	National Bureau of Soil Survey and Land Use Planning, Bhopal
5.	Geology, geomorphology, and lineament data well location data, pre and post monsoon water level data	1:250,000	2021	Central Ground Water Board (CGWB) Raipur, Chhattisgarh
6.	Runoff (2021)	–	2021	Central water Commission (CWC), Bhubaneswar (Kurubhata Gauging station)

### Analytic hierarchy process

The GIS-based MCDA-AHP techniques was used in the present study, which involve and transforms geographical data (input) into the decision (output), where qualitative information on particular themes and attributes is turned into quantitative values by generating a pair comparison matrix using Saaty's scale^51^. Each thematic layers were rated between 1/6 and 4 based on the impacts of these thematic levels and their characteristics on groundwater occurrences (Table 2). A higher score indicates a greater impact on groundwater resources. The scores are diagonally arranged in a Pairwise Comparison Matrix, which has an equal number of rows and columns. The value "1" is diagonally positioned in the matrix, running from the centre to the corner (Table 3).

**Table 2:** Saaty scale based on their impact on Groundwater Potential.

S. no.	Influencing factor	Value	Satty’s scale (fraction)	Satty's scale (decimal)
1.	Rainfall (R)	LOW	1/6	0.17
2.	Curvature (C)		1/5	0.2
3.	Slope (S)		¼	0.25
4.	Drainage density (DD)		1/3	0.33
5.	LULC		½	0.5
6.	Soil texture (ST)		1	1
7.	Lineament (LM)		2	2
8.	Geomorphology (GMY)		3	3
9.	Geology (GY)	HIGH	4	4

**Table 3:** Pairwise comparison matrix.

Criteria	ST	GMY	GY	DD	R	LM	S	C	LULC
ST	1.00	0.33	0.25	3.00	6.00	0.50	4.00	5.00	2.00
GMY	3.00	1.00	0.50	5.00	8.00	2.00	6.00	7.00	4.00
GY	4.00	2.00	1.00	6.00	9.00	3.00	7.00	8.00	5.00
DD	0.33	0.20	0.17	1.00	4.00	0.25	2.00	3.00	0.50
R	0.17	0.13	0.11	0.25	1.00	0.14	0.33	0.50	0.20
LM	2.00	0.50	0.33	4.00	7.00	1.00	5.00	6.00	3.00
S	0.25	0.17	0.14	0.50	3.00	0.20	1.00	2.00	0.33
C	0.20	0.14	0.13	0.33	2.00	0.17	0.50	1.00	0.25
LULC	0.50	0.25	0.20	2.00	5.00	0.33	3.00	4.00	1.00

The paired assessment of parameters in the AHP could often result in certain inconsistency. To assess this, the consistency ratio (CR) was used, and it determined by using the random index scale^51^ and the acquired eigenvalues from the comparison matrix (Eq. 1) (Table 4).^51^ proposed the concept of CR to quantify the amount of consistency of the weight of the parameters. It's the ratio of the consistency index (CI) (Eq. 2) to the random consistency index (RI). The CR is used to demonstrate the correctness of the weights found in the Normalized Pairwise Comparison Matrix (NPCM).

1 $$ {\text{C}}.{\text{R}}. = {\text{C}}.{\text{I}}./{\text{R}}.{\text{I}} $$

where, CI = Consistency Index, RI = Random Consistency Index

**Table 4:** Relative criterion weight and CR values.

Criteria	ST	GMY	GY	DD	R	LM	S	C	LULC	Total	Average	Consistency ratio	Criteria weights (%)
ST	0.09	0.07	0.09	0.14	0.13	0.07	0.14	0.14	0.12	0.98	0.11	9.54	10.88
GMY	0.26	0.21	0.18	0.23	0.18	0.26	0.21	0.19	0.25	1.96	0.22	9.78	21.83
GY	0.35	0.42	0.35	0.27	0.2	0.4	0.24	0.22	0.31	2.76	0.31	9.71	30.71
DD	0.03	0.04	0.06	0.05	0.09	0.03	0.07	0.08	0.03	0.48	0.05	9.16	5.33
R	0.01	0.03	0.04	0.01	0.02	0.02	0.01	0.01	0.01	0.17	0.02	9.22	1.89
LM	0.17	0.11	0.12	0.18	0.16	0.13	0.17	0.16	0.18	1.39	0.15	9.72	15.42
S	0.02	0.04	0.05	0.02	0.07	0.03	0.03	0.05	0.02	0.33	0.03	9.07	3.7
C	0.02	0.03	0.04	0.01	0.04	0.02	0.02	0.03	0.02	0.23	0.03	9.1	2.59
LULC	0.04	0.05	0.07	0.09	0.11	0.04	0.1	0.11	0.06	0.69	0.08	9.34	7.64
Total	1	1	1	1	1	1	1	1	1		Average	9.4	

Consistency index (CI) is evaluated as

2 $$ {\text{C}}.{\text{I}}. = - \lambda {\text{maxn}}/{\text{n}} - {1} $$

where, n = number of factors and λ*max* = average value of the consistency vector.

For a given judgement matrix, a CR value of less than 0.1 is acceptable, and RI is the random index, which is the consistency index of a randomly generated Pairwise Comparison Matrix^11^.

The thematic layers, as well as the sub-criteria weight, were determined and analysed by using a Pairwise Comparison Matrix. The score is assigned to sub-criteria on a scale of 1 to 4 based on favourable conditions and their relevance in detecting the groundwater zone. The most acceptable sub-criteria received a maximum score 4, the least suitable sub-parameters received a minimum score 1, and moderately suitable sub-parameters of the criterion for GPZ identification received an intermediate value (Table 5).

**Table 5:** Percentage Influence and Scale Value of individual themes for the overlay analysis.

S. no.	Parameter	Influence (%)	Feature classes	Feature weight
1.	Geology (GY)	30	Barakar formation	4
			Bastar gneissess	1
			Chandrapur group	3
			Lameta group	3
			Mahadeva formation	4
			Raigarh formation (sandstone)	2
			Talchir formation	4
			Kamthi formation	4
			Raigarh formation	2
			Deccan trap	3
			Chhotanagpur gniessic rocks	1
			Unclassified metamorphic	1
2.	Geomorphology (GMY)	22	Structural plain on Gondawana rocks	4
			Denudational plateau on magmatic and metamorphic rocks	1
			Denudation hills and valleys on proterozoic rocks	2
			Region of denudation hills on Gondwana rocks	4
			Structural plateau on proterozoic rocks	3
			Pediment/pediplain	3
			Structural plain on proterozoic rocks	3
			Region of Plateau	2
3.	Lineament (LM)	15	0–400	1
			400–800	2
			800–1600	3
			1600–2400	4
			2400–3200	4
4.	Soil texture (ST)	11	Gravelly, sand	4
			Sandy clay loam, clay loam	4
			Silt loam, loam	3
			Loamy sand, sandy loam	2
			Clay, silt clay	1
5.	LULC	8	Agricultural land	4
			Shallow water body	4
			Deep water body	4
			Dense forest	3
			Open forest	3
			Fallow land	4
			Barren land	2
			Scrubland	3
			Settlement	1
6.	Drainage density (DD)	5	0–1 km/km^2^	4
			1–2 km/km^2^	3
			2–3 km/km^2^	2
			3–4 km/km^2^	1
7.	Slope (S)	4	0–2%	4
			2–4%	4
			4–6%	3
			6–8%	2
			8–35%	1
8.	Curvature (C)	3	Concave	4
			Linear	3
			Convex	2
9.	Rainfall (R)	2	1200–1300 mm	2
			1300–1400 mm	3
			1400–1500 mm	4
			1500–1600 mm	4
			1600–1750 mm	4

The thematic layers are then reclassed using the weightage values obtained after being transformed to 30 × 30 m cell size. The weighted overlay analysis (WOA) is a technique that allows users to address spatially complicated site suitability concerns using common measures of several inputs and accordingly GZPs were identified. In the WOA tool, all reclassified raster maps overlayed and accordingly weights were assigned. Finally, the cell score of each input raster is multiplied by the weighted values of each raster layer (Eq. 3). The generated raster layer was divided into four groups of GPZs with the same range of given weights based on the United Nations' Food and Agricultural Organization (FAO) recommendations.

3 $$S=\sum_{i=1}^{n}(wi.xi)$$

where, S is total GPZ score, *wi* denotes weight of GPZ criteria, *xi* Indicates sub-criteria score of i GPZ criteria, and *n* represents total number of GPZ criteria.

**Figure 2:**
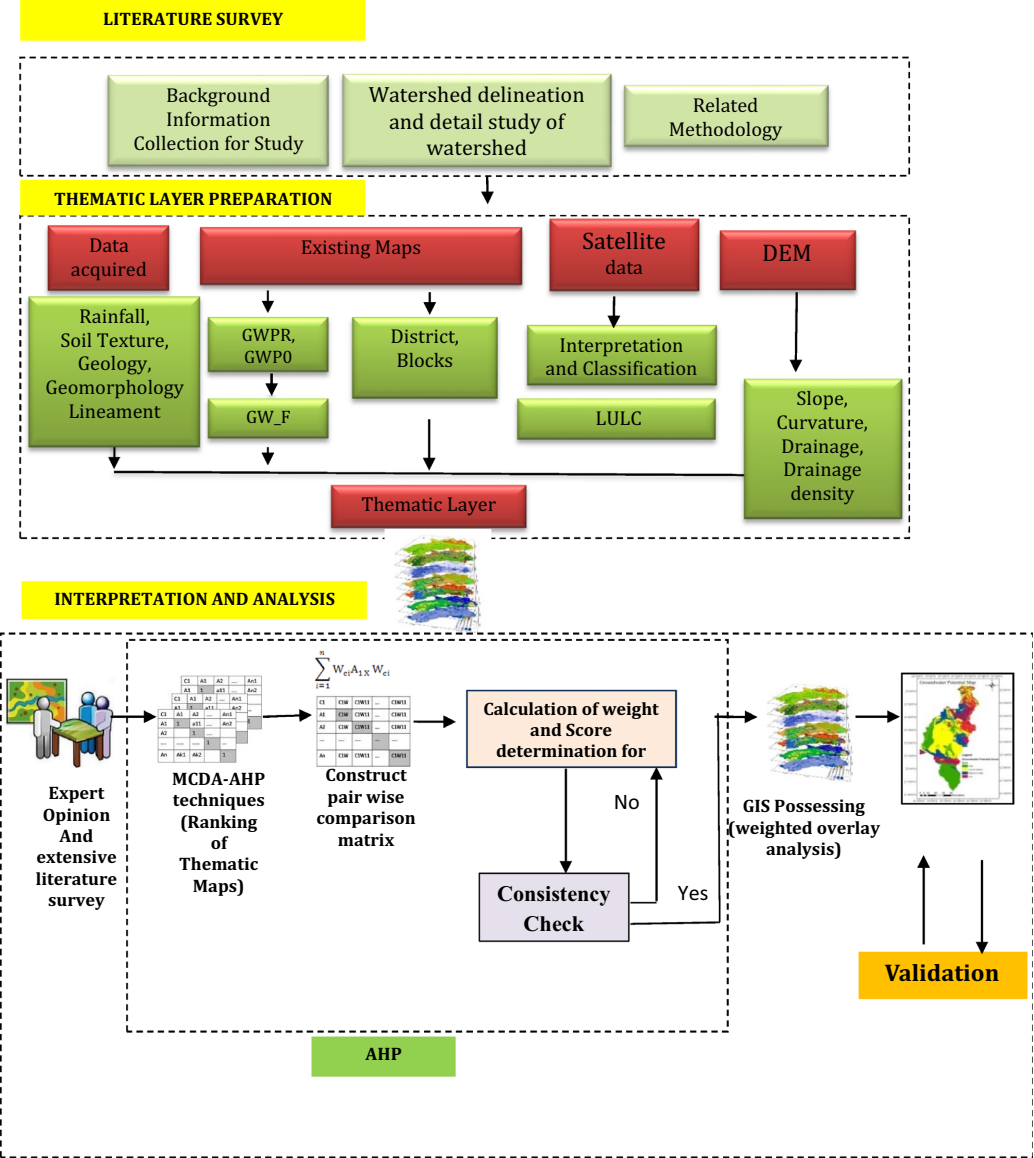
Flowchart of the methodology.

Finally, the overlay analysis is used to generate the groundwater potential map, which is then validated using fluctuation data. Figure 2 depicts the methodological flowchart of the present study.

### Appropriate locations for artificial recharge structures

The artificial recharge structures were suggested based on the topography, land-use class, slope, aspect, and soil type^76^. In the present study, the artificial recharge structures (percolation tank, check dam and farm pond) have been proposed based on the recommendations of the Indian National Committee on Hydrology (INCOH)^76^. To select the suitable structures, the different selection criterias like dimensions and its applications were discussed in Tables 6 and 7.

**Table 6:** Site selection criteria for the structures.

Type of structure	Maximum water level (m)	Slope (%)	Soil texture	Rainfall (in mm)	Permeability	Storage loss	Land use	Drainage
Check dam	4–5	< 15%	Sandy clay loam	< 1000 mm	Low	Low	Barren, scrubland	Higher order i.e., > 3rd order
Percolation tank	6–7	< 10%	Silt loam, clay loam	< 1000 mm	High	Moderate-high	Barren, Ss	2nd to 3rd order
Farm pond	2–2.5	< 5%	Sandy clay loam, silt loam	> 200 mm	Low	Moderate-low	Scrubland, moderately cultivate	1st order

**Table 7:** Specifications for the structures.

Type of structure	Applications	Required site condition	Dimensional parameters
Percolation tank	Recharge to aquifer and surface storage for restricted period. May be used for limited irrigation, livestock and domestic	Permeability high, well defined broad stream channel and presence of intersecting fractures	3–5 m high earthen bund, 5000–10,000 m^3^ effective storage, shallow cutoff, provision for spillway, silt trap barrier in the upstream
Check dam	Surface storage, Restricted irrigation, and domestic needs	Well defined straight stream channel with level banks, adequate catchment and rocky riverbed without any fractures	2–4 m height of masonry structure, 5000–7000 m^3^ effective storage, partial treatment to foundation for leakage/seepage, provisions for overflow
Farm pond	For livestock storage, a restricted irrigation	Narrow elongated depression with gentle slope and small catchment area	1–2 m high elongated earthen embankment, 2000–5000 m3 storage, shallow foundation

### Estimation of available volume of subsurface storage

The total volume of subsurface storage was estimated based on the thickness of unsaturated zone (within 10 mbgl). And it was assumed that the volume of unsaturated strata which will recharge and store the groundwater will be 40% of the total volume of subsurface storage^77^.

### Availability of surplus water for recharge

Surplus runoff was account as 40% of the total runoff generated from Mand catchment for the artificial recharge of aquifers^77^.

### Research involving human participants and/or animals

This article does not contain any studies involving animals performed by any of the authors. This article does not contain any studies involving human participants performed by any of the authors.

## Results and discussion

### Drainage density map

**Figure 3:**
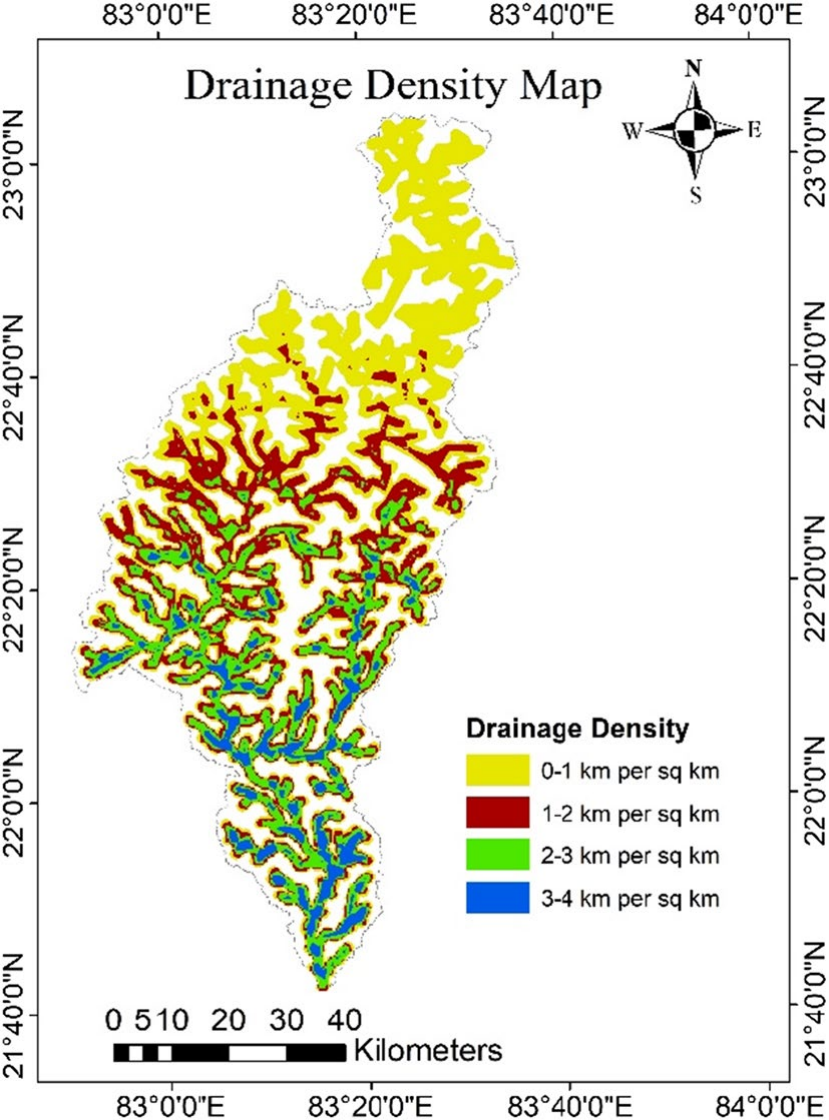
Drainage density map of the catchment (this figure was generated using ArcGIS 10.4 http://appsforms.esri.com/products/download/index.cfm?fuseaction=download.main&downloadid=1932).

The drainage density (DD) of the study was estimated by using the total stream length (11,651.15 km), and the total catchment area. From the analysis it was observed that the study area was having a total of 20,203 streams. The DD of the study area were ranges from 0.75 to 4 km/km^2^. Further, the DD was divided into four classes as extremely high (3–4 km/km^2^), high (2–3 km/km^2^), medium (1–2 km/km^2^), and low (0–1 km/km^2^) (Fig. 3). A total of 58% of the area falls into the low (0–1 km/km^2^) DD category, and 12% of area falls into the very high (3–4 km/km^2^) DD category. In the present study, higher weights were assigned to low DD regions and lower weights were assigned to high DD regions. The Low DD indicates more rain water infiltration and contributes to groundwater potential, whereas higher values of DD indicate the high surface runoff and less infiltration^62^.

### Rainfall map

**Figure 4:**
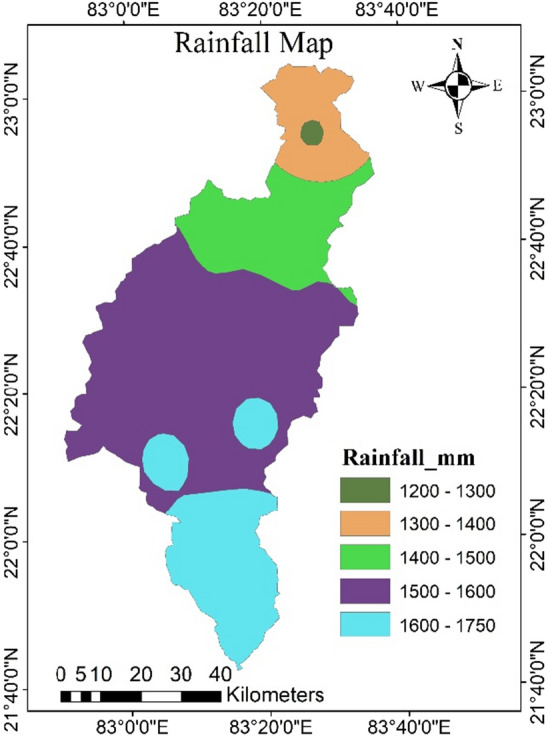
Spatial distribution of rainfall in the catchment (this figure was generated using ArcGIS 10.4 http://appsforms.esri.com/products/download/index.cfm?fuseaction=download.main&downloadid=1932).

Rainfall is the principal source of groundwater recharge in the study area, and almost 85% of it receives during the southwest monsoon season. The rate of infiltration of runoff water is directly affected by rainfall distribution and slope gradient, increasing the probability of potential groundwater zones. The annual rainfall of the study area was ranges from 1291 to 1734 mm, and further it was divided into five classes as very low (1200–1300 mm), low (1300–1400 mm), moderate (1400–1500 mm), high (1500–1600 mm), and very high (1600–1750 mm)^45^ Fig. 4). In the present study, the high rainfall classes were given a high weight of 4, and vice versa for the AHP analysis. In the study region, the northern part receives the least amount of rainfall (about 20% of the total area), whereas the southern part receives highest amount of rainfall.

### Soil texture map

**Figure 5:**
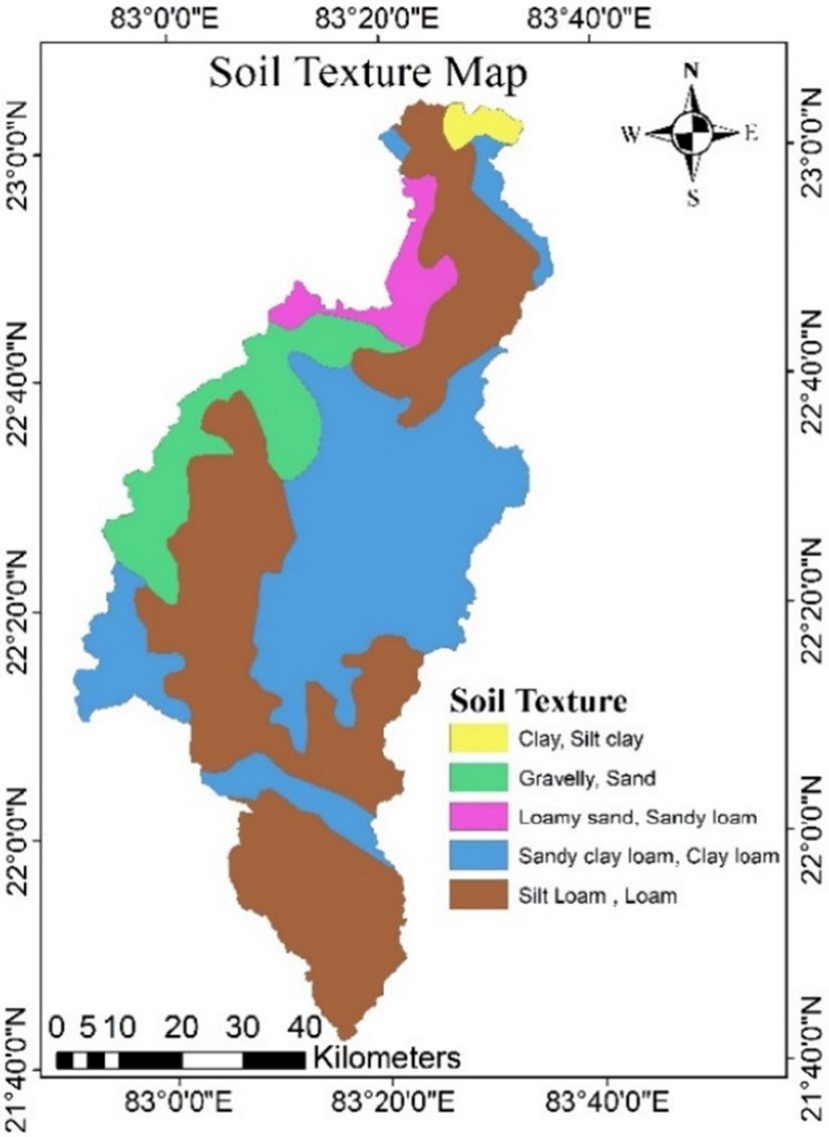
Soil texture map of the study area (this figure was generated using ArcGIS 10.4 http://appsforms.esri.com/products/download/index.cfm?fuseaction=download.main&downloadid=1932).

Soil texture is an important geomorphic component to determine the GPZ. It influences the soil structure, void ratio, porosity, and permeability of the soil^4^. The dominant soil group of the study area was silt loam and loamy texture (red and yellow colored) soil, which having the moderate runoff potential, moderate rate of water transmission (0.15–0.30 in/hr), and moderate infiltration rate (3.81–7.62 mm/hr) (Fig. 5, Table 8). The soil texture (gravel, sand, and sandy loam) having high infiltration rate (7.62–11.43 mm/hr), and high-water transfer (0.30 in/hr) are given high weightage values, indicating strong groundwater potential. And soil texture (clay, silt and loam) having low infiltration rate (0–3.81 mm/hr), and low water transmission (0–0.15 in/hr) capacity are given lower weightage due to their low groundwater potential (USDA-SCS Soil classification).

**Table 8:** Areas under different soil texture.

S. no.	Soil type	Soil name	Soil texture	Area (km^2^)	Total area (%)
1.	Red gravelly soil	Alfisols	Gravelly, sand	585.41	10.98
2.	Red sandy soils	Alfisols	Sandy clay loam, clay loam	2014.65	37.78
3.	Shallow black soils	Inceptisols	Clay, silt clay	62.24	1.17
4.	Lateritic soils	Ultisols	Loamy sand, sandy loam	215.99	4.05
5.	Red and yellow soils	Ultisols	Silt loam, loam	2453.87	46.02

### Slope map

**Figure 6:**
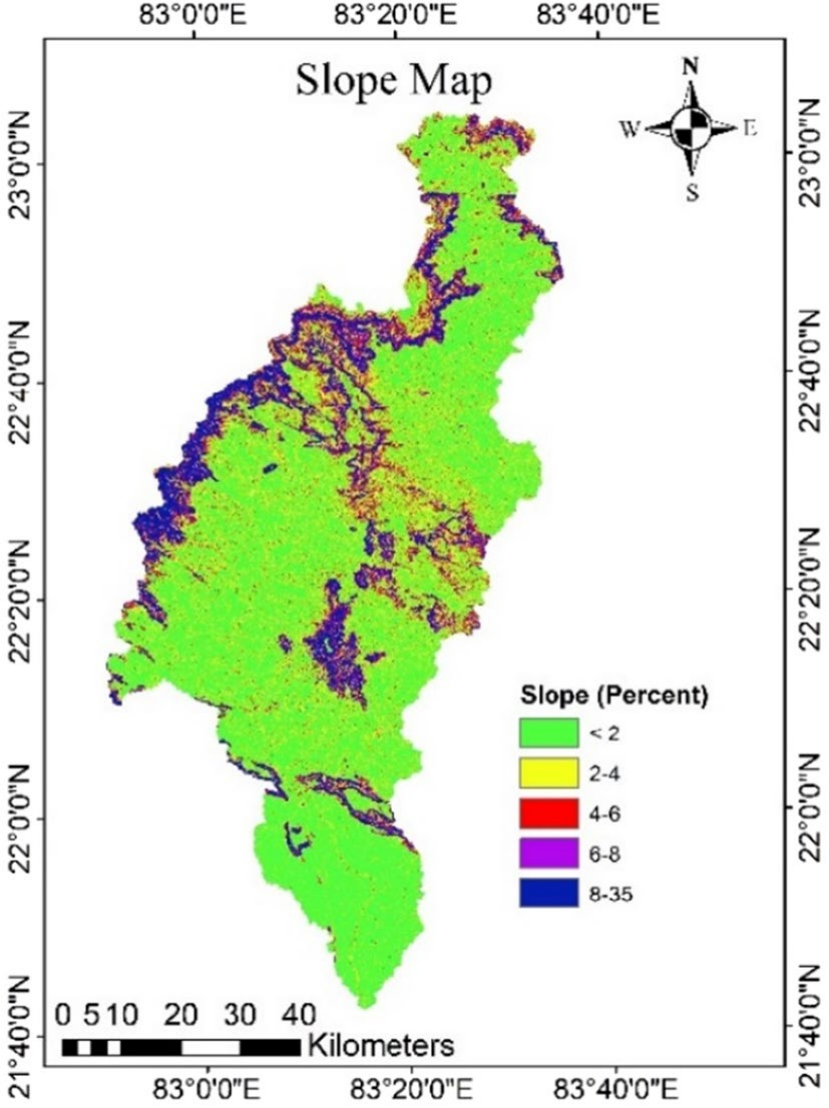
Slope of the study area (this figure was generated using ArcGIS 10.4 http://appsforms.esri.com/products/download/index.cfm?fuseaction=download.main&downloadid=1932).

The slope of the study area was divided into six categoriesas level (0%), nearly level (0–2%), very gently sloping (2–4%), gently sloping (4–6%), slightly moderate sloping (6–8%), moderately sloping (8–10%), strongly sloping (10–14%), steep sloping (14–16%) and very steep sloping (> 16%) (Fig. 6). The slope map of the catchment illustrates a complicated topography with undulations and uneven slopes. The majority of the watershed contains almost flat to moderately sloping fields, which can be regarded excellent groundwater recharge sites since surface water has more time to infiltrate and accordingly higher weights were given. The catchment has a region with strong to severe slope, which is bad for groundwater recharge as surface water does not have time to infiltrate through the soil surface.

### Geology map

**Figure 7:**
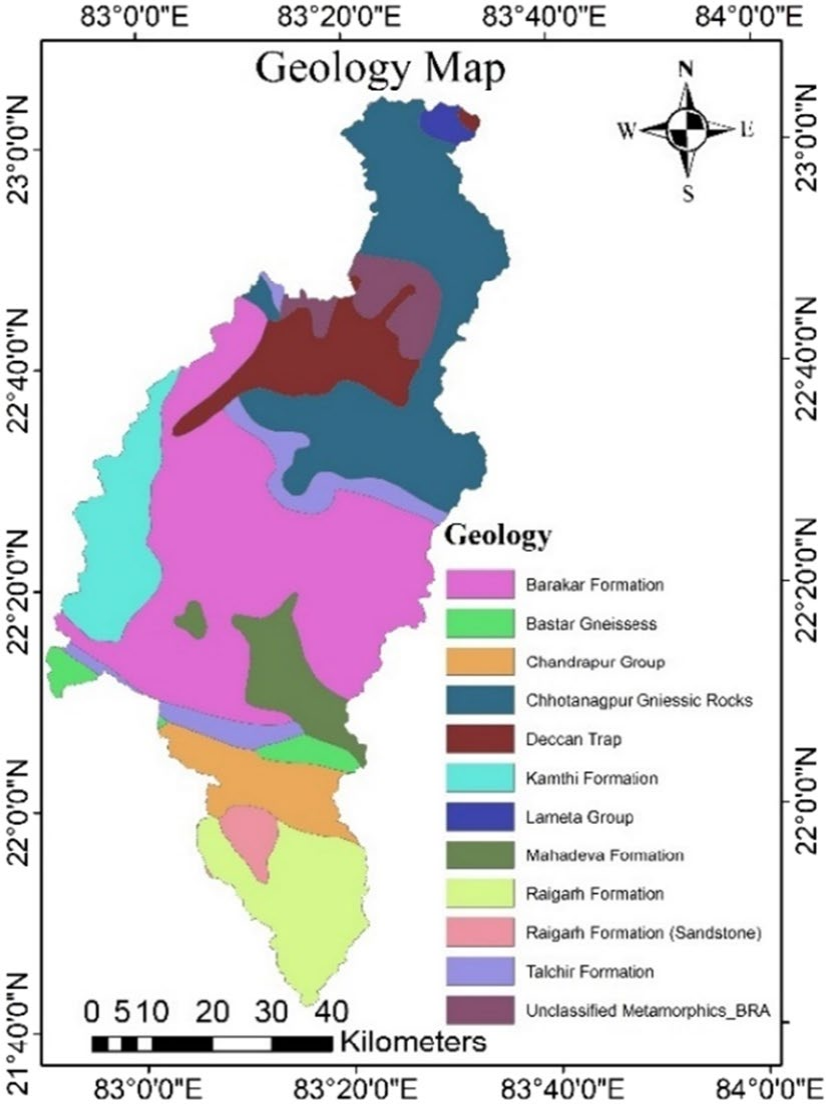
Geology map of the study area (this figure was generated using ArcGIS 10.4 http://appsforms.esri.com/products/download/index.cfm?fuseaction=download.main&downloadid=1932).

The type of groundwater occurrences and their distribution is heavily influenced by geology. The Mand catchment’s predominant geology is Gondwana rocks, 46% of its area followed by Chhotanagpur gneissic complex (26%) and Chhattisgarh supergroup, which includes the Raigarh and Chandrapur formations (15.25%) (Fig. 7). Talchir, Barakar, Kamthi and Mahadeva formation are the Gondwana rocks of the region. The Barakar Formation covers the majority of Gondwana (32.85%) followed by Kamthi (7.47%) (Table 9). The Barakar is the study area's only coal-bearing deposit both in the shallow and deeper zones. Kamthi are the newest members and are mostly represented by sandstones and shales which are iron-rich and filthy to brownish in colour. The Talchir formation’s lithology include shale, sandstone, and boulder bed. Because Gondwana is made up of sediments, it is assigned the highest weighting because of the increased likelihood of groundwater occurrences owing to its lithology.

**Table 9:** Geology formation and area distribution.

S. no.	Geology	Area (in sq.km.)	Area (%)
1.	Barakar formation	1751.48	32.85
2.	Bastar gneissess	105.12	1.97
3.	Chandrapur group	280.51	5.26
4.	Lameta group	43.53	0.82
5.	Mahadeva formation	260.17	4.88
6.	Raigarh formation(sandstone)	81.17	1.52
7.	Talchir formation	272.11	5.10
8.	Kamthi formation	398.11	7.47
9.	Raigarh formation	461.48	8.65
10.	Deccan trap	393.774	7.39
11.	Chhotanagpur gniessic rocks	1089.125	20.43
12.	Unclassified metamorphic	195.489	3.67

The crystalline and metamorphic rocks, which are part of the Chhotanagpur gneissic complex, are mostly found at the northern edge of the region. The gneissic rocks of Chhotanagpur are mostly quartz mica schist and quartzite with granite gneiss, intruded by granite and dolerite^72^. They are given the least weight because of their lithology, which has a low water transmissivity.

The phreatic aquifer in the Chhattisgarh supergroup was given moderate weighting since the area is good for groundwater development due to its good production potential.

### Geomorphology map

**Figure 8:**
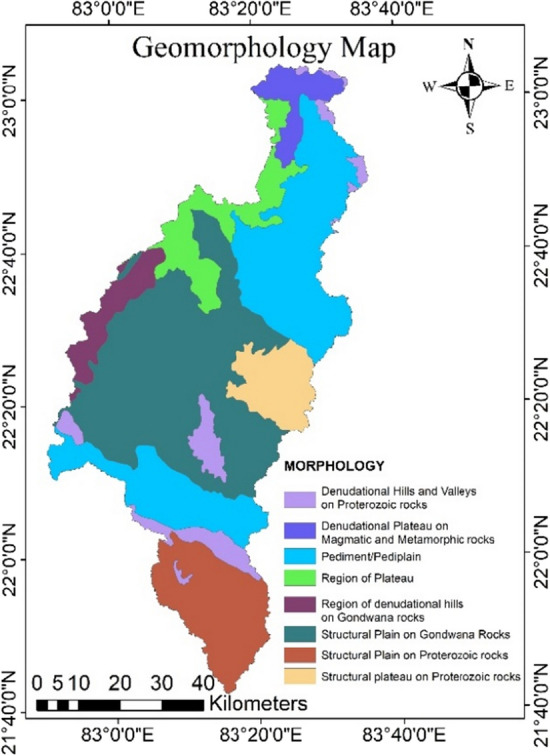
Geomorphology of the study area (this figure was generated using ArcGIS 10.4 http://appsforms.esri.com/products/download/index.cfm?fuseaction=download.main&downloadid=1932).

Geomorphology is the important element for understanding the presence, potential, and flow of groundwater resources due to its tectonic activity and denudational processes. The structural plains on Gondwana rocks covers 46% of the area followed by pediment/ pediplains (26%) and structural plains and plateaus on proterozoic rocks (21%) dominates the Mand catchment (Fig. 8). The structural plains on Gondwana rocks have been assigned the greatest weighting; due to its sedimentary origin, it serves as an outstanding groundwater recharge source^6^. The pediment/ pediplain complex located in the north-eastern and southern parts of the catchment which is made up of weathered colluvium material or gravel; it serves as a significant groundwater recharge source in the catchment and is thus given more weightage.

The structural plains and plateaus on proterozoic rocks covers southern and eastern portion respectively are given moderate weightage due to the high transmission rates of alluvium deposits on a gentle slope and are frequently connected with well potentials. The least weighted value was provided to the denudational plateau on magnetic and metamorphic rocks since they have more surface runoff than recharge due to poor water transmission.

### Lineament map

**Figure 9:**
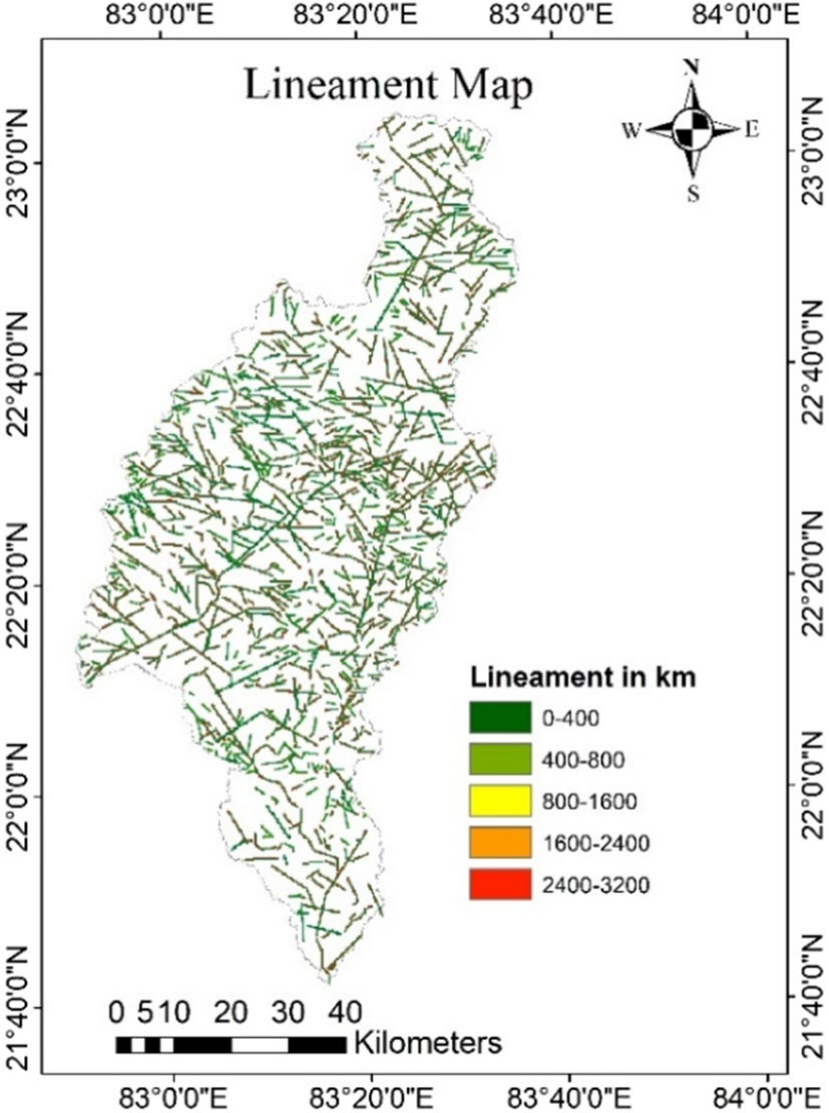
Lineament map of the study area (this figure was generated using ArcGIS 10.4 http://appsforms.esri.com/products/download/index.cfm?fuseaction=download.main&downloadid=1932).

A lineament is a linear feature i.e., fault and fracture in a landscape that represents the geological structure beneath it. Lineaments enhances secondary porosity and permeability that are crucial in terms of hydrogeology because they provide pathways for groundwater circulation^40^ (Fig. 9). As a result, these characteristics define the GPZs^58^. The possibility of a potential groundwater area decreases with decreasing lineament number and increases with increasing number of lineaments^57^.

Due to extensive lineaments, the lineament map suggests that the southern and western parts of the study region are very appropriate for groundwater recharge. Based on lineaments density, the northern and some central parts of the state have less potential for groundwater recharge since density declines from the south to the centre and then to the north. Table 10 lists the different lineament structures as well as their corresponding lengths.

**Table 10:** Various lineament structures and their respective lengths.

S. no.	Lineament structures	Length (in kms.)
1.	Fault	550.52
2.	Fracture/lineament	2196.15
3.	Intrusive bodies (lines)	278.93
4.	Shear zone	8.04

### Land use and land cover (LULC) map

**Figure 10:**
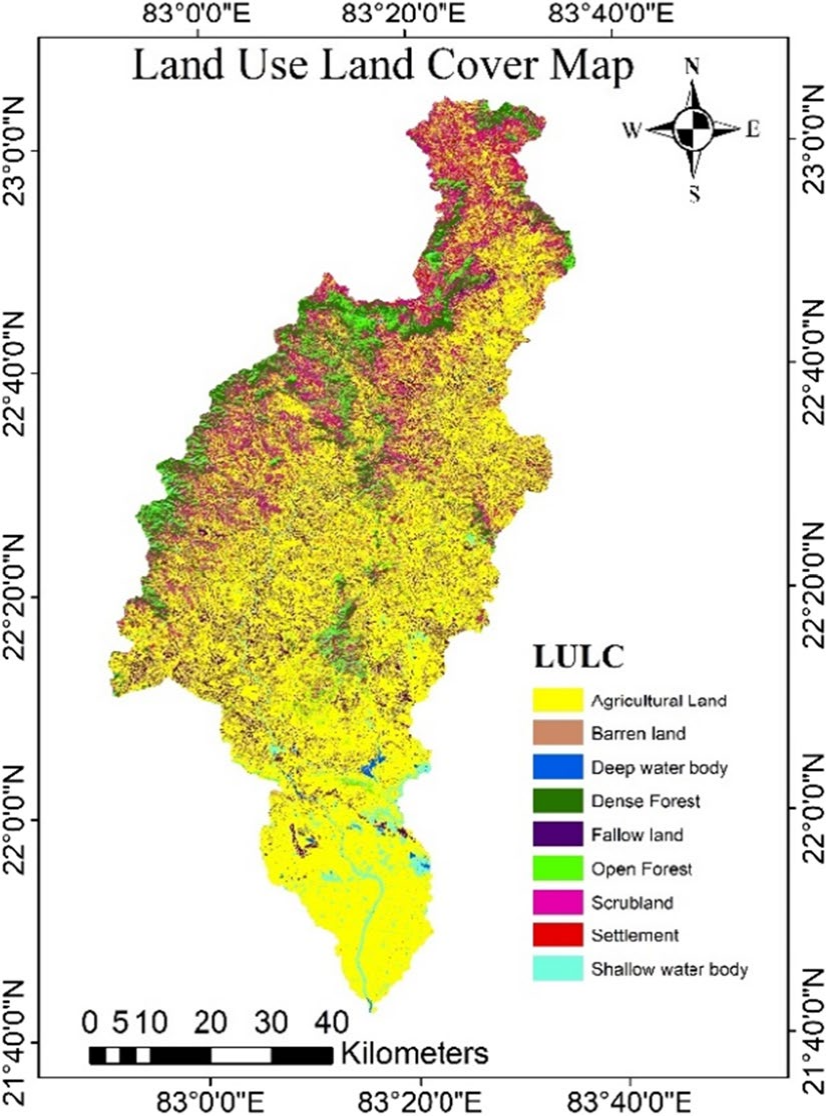
LULC map of the study area (this figure was generated using ArcGIS 10.4 http://appsforms.esri.com/products/download/index.cfm?fuseaction=download.main&downloadid=1932).

The hydrogeological, geo-hydroclimatic, and water cycles processes like runoff, infiltration, and evapotranspiration are significantly impacted by LULC and ultimately affect groundwater recharge. The LULC of the study area was divided into nine classes such as agricultural land, fallow land, settlement, dense forest, open forest, barren land, shallow water body, deep water body, and scrubland (Fig. 10, Table 11) Forests and scrubland predominate in the upper part of the catchment, whereas the middle and lower part of catchment having predominately agricultural land, settlement and water bodies. The land use land cover was validated using google satellite image, ground truthing data, and kappa coefficient. The kappa coefficient (Kp) was calculated based on the method discussed by other researchers^78,79^. The Kappa coefficient ranges from 0 to 1, and a higher coefficient value indicates more accuracy^80,81^.

**Table 11:** LULC classes and area distribution.

S. no.	LULC classes	Area (sq. km)	Area (%)
1.	Agricultural land	3099.84	58.14
2.	Shallow water body	185.36	3.48
3.	Deep water body	15.73	0.30
4.	Dense forest	329.87	6.19
5.	Open forest	316.77	5.94
6.	Fallow land	540.01	10.13
7.	Barren land	19.97	0.37
8.	Scrubland	718.70	13.48
9.	Settlement	105.82	1.98

In overall the agricultural land covers almost 58.14% of the total catchment area, which is followed by the scrubland and fallow land. The classification's overall accuracy was found to be 89.25%, and the Kappa coefficient to be 0.91. The Chhattisgarh districts of Bastar, Dhamtari and Korba observed similar outcomes^82–84^.

Amongst the all LULC, water bodies were given the highest score as 4 since in the research region carry a substantial volume of water throughout the year. Similarly, agricultural land and dense forest area was also given highest score as 4 as the roots of the trees loosen the soil and increased the water holding capacity and percolation. Fallow land, barren land, and settlement were given moderate to low score as 3 to 1 based on its water transmission and water holding capacity.

### Curvature

**Figure 11:**
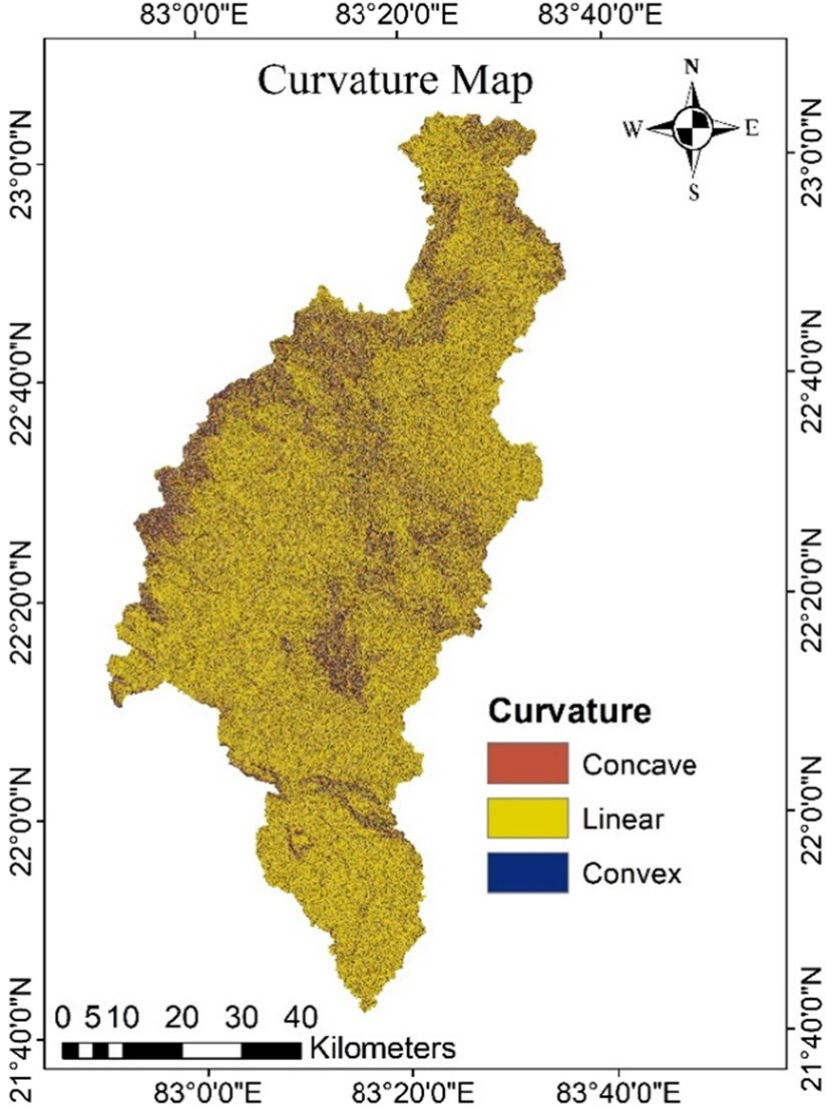
Curvature map of the study area in Arc-GIS 10.4 (http://appsforms.esri.com/products/download/index.cfm?fuseaction=download.main&downloadid=1932).

The curvature of hill slopes, which represents the morphology of the regional topography, is a significant factor to consider in the case of groundwater hydrology and terrain instability. It is the shape of the surface profile, which might be concave, linear, or convex^40^. The shapes and curvatures of a slope have a strong influence on the dynamics of surface and subsurface hydrology, as well as the development and accumulation of soil. In comparison to convex slopes, the soil thickness is greater on concave slopes. Because surface and subsurface water collect on the concave slope, increasing pore water pressure during storms and severe downpours, it is given more weight for groundwater potential. A quick runoff occurs on a convex slope, avoiding water storage and resulting in less weight given to groundwater potential. Convex slopes include intervening hills and side slopes. Concave features include erosional landforms such as gullies. Planar landforms are those that fall between the concave and convex slope categories. In the Mand catchment, the curvature values range from 8.51 to + 9.08 and the Fig. 11 is depicting curvature map of the area. A positive curvature value indicates that the surface is convex, whereas a negative curvature value indicates that the slope is concave. Linear surfaces are given the value zero.

### Identification of groundwater potential zones

The above mentioned nine parameters were considered for identification of groundwater potential zones. These parameters were used in the AHP approach and accordingly weights were assigned based on the Pairwise Comparison Matrix. The CR and CI was found as 0.05 and 0.094, respectively for high GPZs.

**Figure 12:**
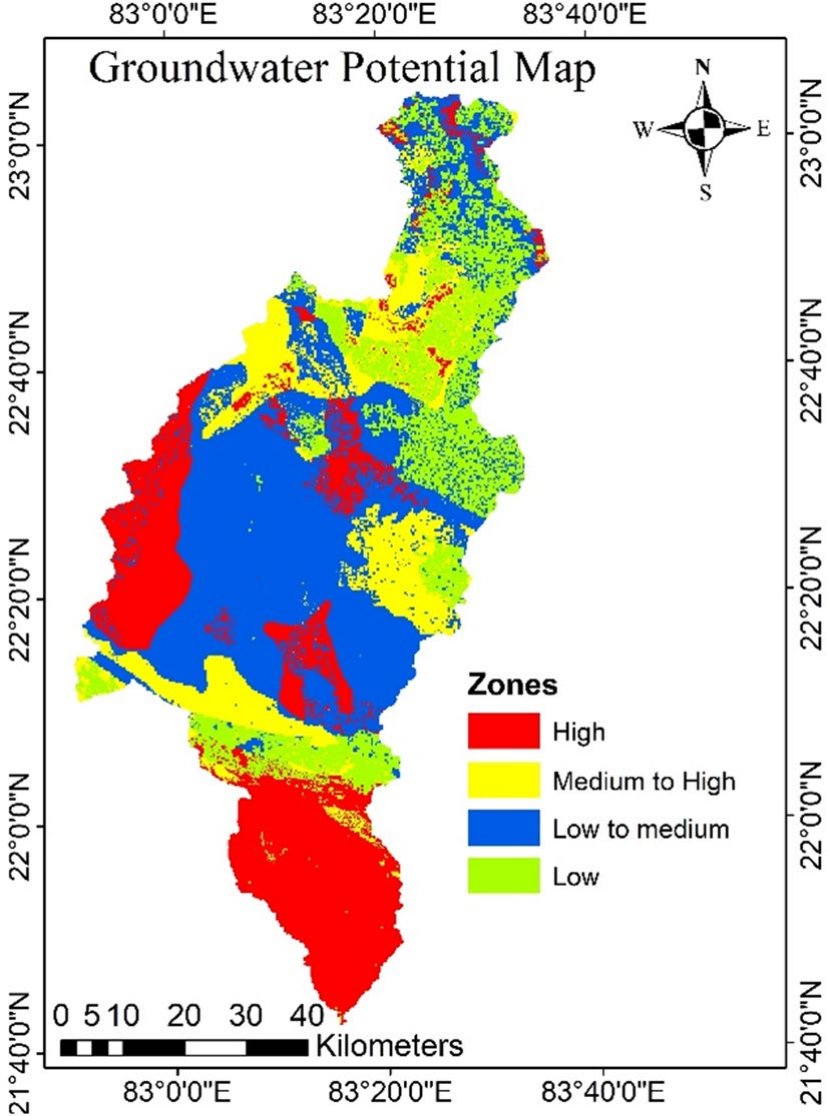
Ground water potential zones of the study area in Arc-GIS 10.4 (http://appsforms.esri.com/products/download/index.cfm?fuseaction=download.main&downloadid=1932).

The groundwater potential zones (GPZs) of the study area were divided into four groups such as Low, low to medium, medium to high, and extremely high GPZs, which covers the area of 18.05, 37.88, 18.18, and 25.89%, respectively (Fig. 12, Table 12). The very high potential zone was found in the areas like Kharsia, Raigarh, Korba, and some parts of Dharamjaigarh block, and the low GPZs were found in Lundra, Batauli, Pathalgaon, Sitapur blocks. Due to the availability of loamy textured well-drained soil, high intensity of rainfall, presence of lineament, gentle slope, concave curvature, favourable geological formation, and vast agricultural land with excellent infiltration capability, the results show that the excellent GPZ was concentrated in the southern, western, and small area in the central part of the catchment. And existence of poorly drained soil, less rainfall, metamorphic rock underneath, smaller number of lineaments and convex curvature the northern and north-west section of the study region has a very poor or low potential for groundwater recharge. Comparable results were reported for the Karun watershed of Chhattisgarh^76^ and in some identical watersheds with similar methodology^20,40,45^.

**Table 12:** GPZs and area distribution.

S. no.	GPZs	Area in sq.km	Area (in %)
1.	Very low	962.44	18.05
2.	Low to medium	2019.92	37.88
3.	Medium to high	889.06	18.18
4.	Very high	1380.42	25.89

## Validation

**Figure 13:**
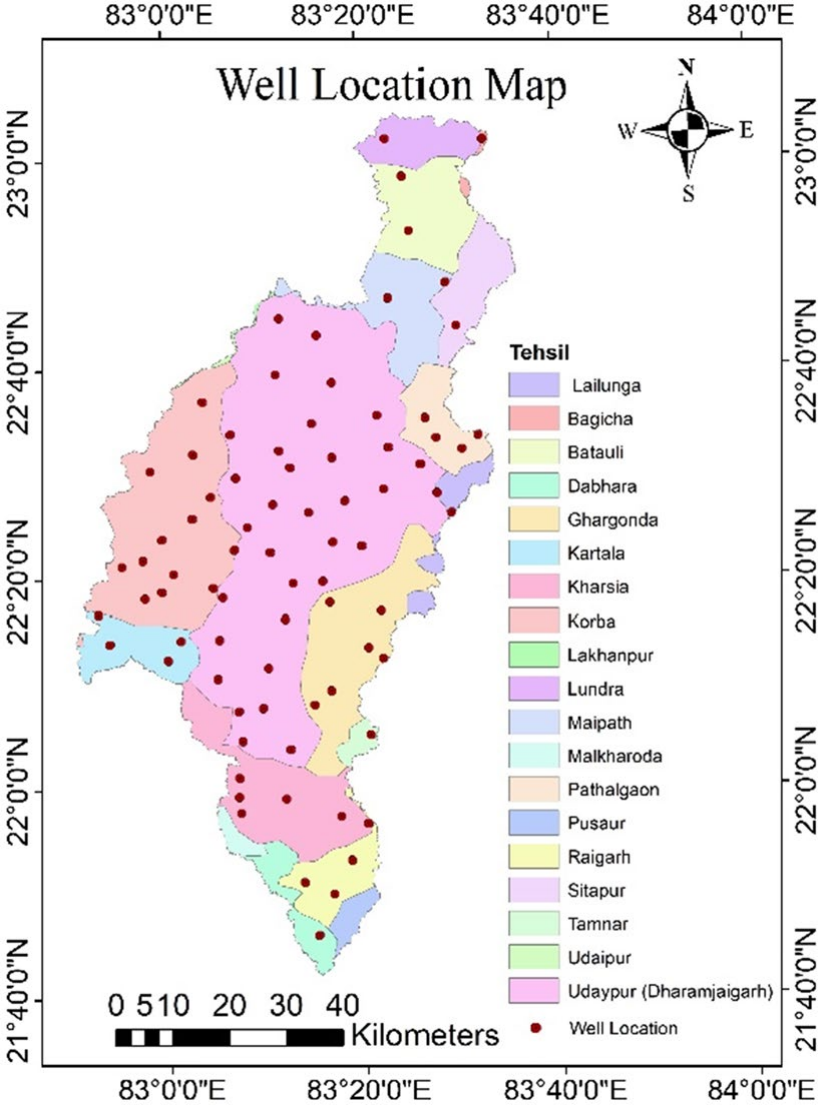
Well location map (this figure was generated using ArcGIS 10.4 http://appsforms.esri.com/products/download/index.cfm?fuseaction=download.main&downloadid=1932).

**Figure 14:**
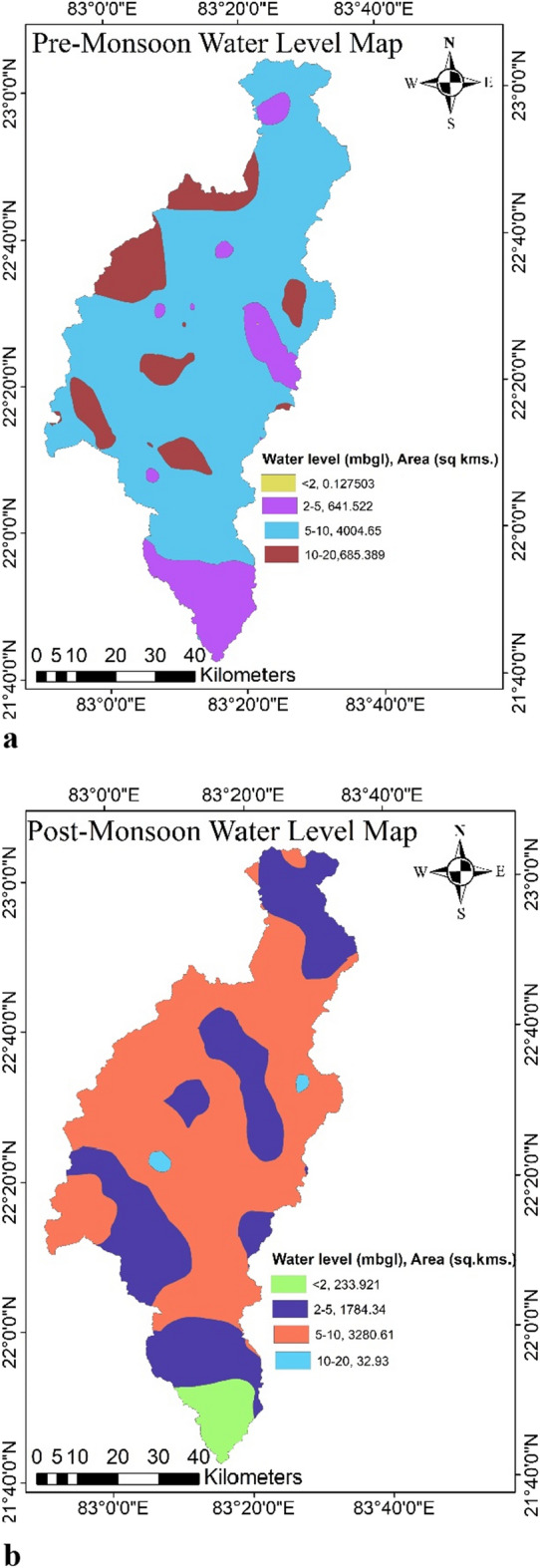
(a) Pre monsoon water level in the study area, (**b**) post monsoon water level in the study area (this figure was generated using ArcGIS 10.4 http://appsforms.esri.com/products/download/index.cfm?fuseaction=download.main&downloadid=1932).

The validation of the estimated GPZs was done by comparing them with the groundwater fluctuation map calculated using observation well (Fig. 13) data obtained from the Central Groundwater Board's observation well data (CGWB). In this study, a total of 79 observation wells data were taken into the consideration. The groundwater fluctuation map was generated by using pre-monsoon (April) and post-monsoon (December) mbgl (metre below ground level) water level data (Fig. 14a,b). In general, areas with greater water level fluctuation have low groundwater potential, while those with less water level fluctuation typically have high groundwater potential^45,62^.

**Figure 15:**
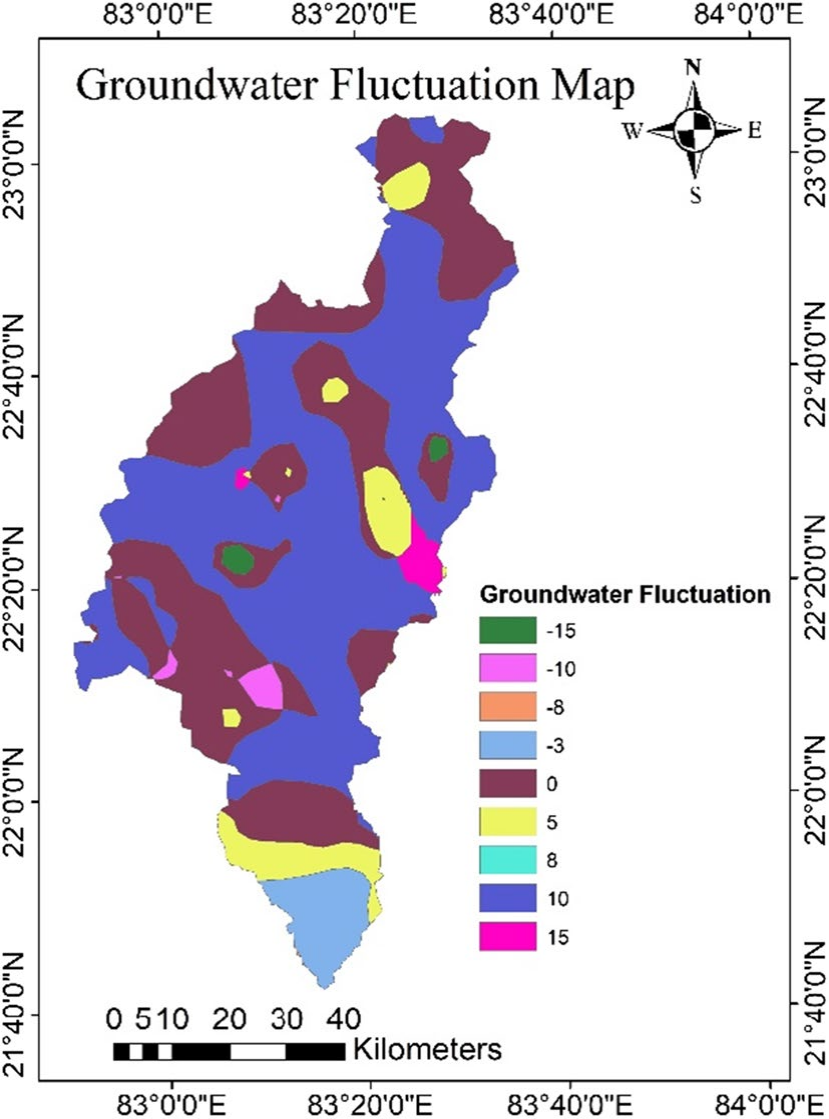
Groundwater fluctuation map (this figure was generated using ArcGIS 10.4 http://appsforms.esri.com/products/download/index.cfm?fuseaction=download.main&downloadid=1932).

**Figure 16:**
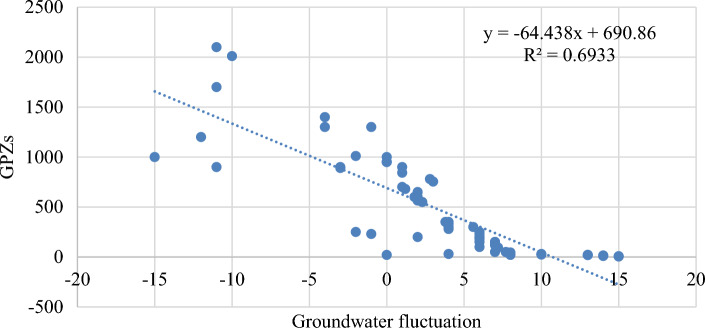
Relationship between grounder potential zones and groundwater fluctuation.

During the pre-monsoon season the groundwater table fluctuates from 1.9 to 19.95 mbgl, with an average of 10.40 mbgl, this may be due to the extraction of groundwater for irrigation. The shallowest water table depth was found in the southern part of the catchment, while the deep-water table depth is found in the western part. The groundwater table fluctuates during the post monsoon was ranges from 2.09 and 18.30 mbgl, with an average of 6.42 mbgl. From the groundwater fluctuation map, it was observed that the pathalgaon block has the deepest water depth, whereas Dabhara and Pusaur have the shallowest depth. The groundwater fluctuation map (Fig. 15) shows that the southern and some north-western parts of the study area have low groundwater fluctuation, indicating high water potential zone. Whereas the western and some central parts of the study area have more water table fluctuations, indicating low groundwater potential zone. The scatter plots in Fig. 16 reveal a negative relationship between groundwater fluctuation and groundwater potential. From the study result it can infer that the groundwater potential map is very accurate when compared to groundwater fluctuation maps and may be utilised for groundwater resource management in the Mand catchment. It was discovered that wells in low GPZs had a water yield capability of 10–50 L per minute (lpm). whereas in medium, medium to high, and high GPZs, had the water yield capacity as 50–100 lpm, and 100–200 lpm, respectively. It can be inferred from the study that the GIS and AHP-based methodologies for delineating GPZs used here are a viable way for river basin-based planning and development in tropical and sub-tropical regions with a variety of geo-environmental scenarios.

## Development of management plan for low and low to medium groundwater potential areas

**Figure 17:**
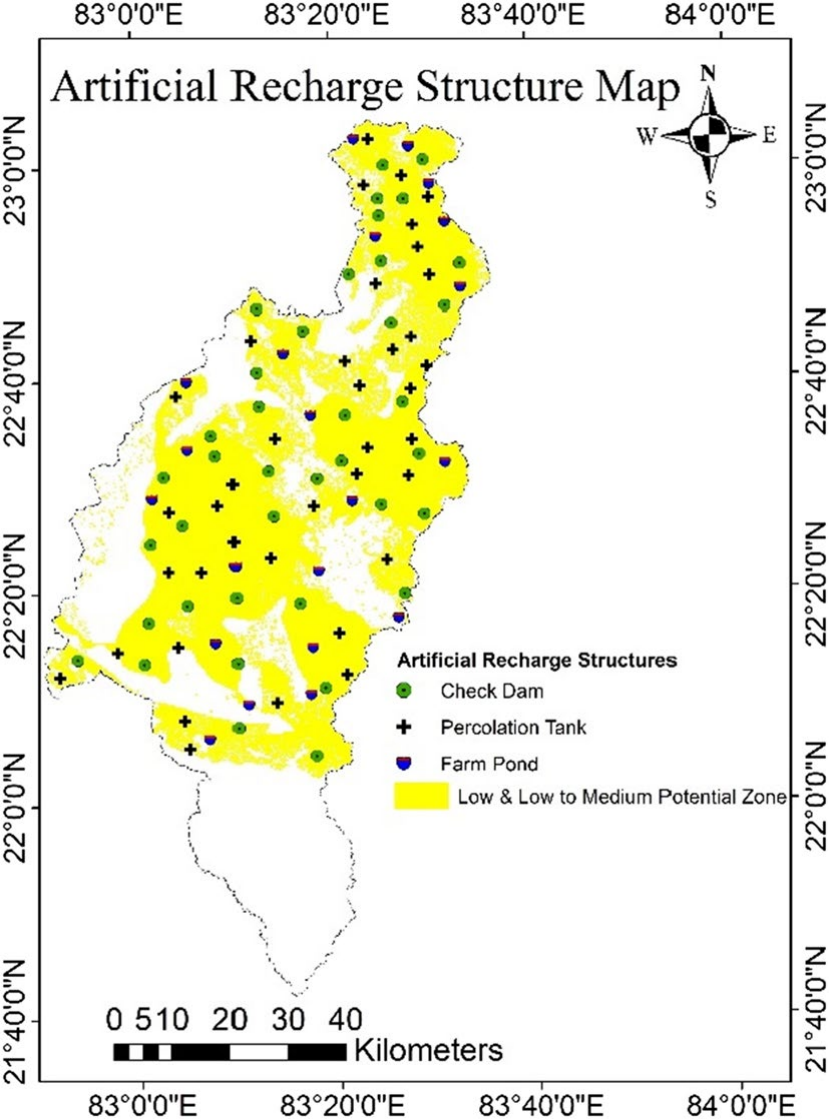
Artificial recharge structures Map in Arc-GIS 10.4 (http://appsforms.esri.com/products/download/index.cfm?fuseaction=download.main&downloadid=1932).

The high groundwater potential zone implies that the availability or bearing capacity of capacity of groundwater is high in the area. And the management plan is needed for the low and low to medium potential zones for development or recharging of groundwater. The type of structures to be constructed for groundwater management was selected based on the GPZs map, depth to water level map, and topography of the study area. Different structures like percolation tanks, check dams, and farm ponds were proposed for artificial recharge and storing of water in the low and low to medium GPZs. Total number of percolation tanks, check dams, and farm ponds as 36, 39, and 21, respectively (Fig 17).

In the Mand catchment, the volume of unsaturated strata that will recharge and store groundwater is 21.33 km^3^, or 40% of the total subsurface storage^77^. The total amount of runoff generated by the Mand catchment was account as 5.07 km^3^. Out of the total runoff 2.03 km^3^ (40%) was deemed as excess for artificial recharge^77^. This predicted runoff can be accommodated by the computed subsurface storage capacity, which will raise groundwater levels in the low and low to medium GPZs.

## Conclusion

The current study assessed the groundwater potential using AHP and GIS methodologies in the agriculture dominated Mand catchment in the middle Mahanadi Basin. To map the GPZs of the research region, nine criteria were weighted and overlaid into the ArcGIS 10.5 environment: geology, geomorphology, curvature, slope, LULC, drainage density, lineament, soil, and rainfall. The groundwater potential shows that 37.88% of the area is in the low to medium potential zone, 18.18% is in the medium to high potential zone, 25.89% is in the very high potential zone covering the southern and western parts, and only 18.05% is in the very low GPZs covering the northern and north-western parts.

The research considers initiatives to manage extensive water usage by seasonal surface water storage and recharging it with seasonal runoff, as well as modification of irrigation methods in places with low and low to medium potential recharge. The runoff of the study area can be accommodated by the computing subsurface storage capacity, which will raise groundwater levels in the low and low to medium GPZs. The computed subsurface storage capacity (21.33 km^3^ or 40% of the total subsurface storage area) can accommodate the runoff (2.03 km^3^ or 40% of total runoff), raising groundwater levels in the low and low to medium GPZs. According to the study results, various groundwater recharge structures such as farm ponds, check dams and percolation tanks were suggested in appropriate locations of the Mand catchment to boost groundwater conditions and meet the shortage of water resources in agriculture and domestic use. Total number of percolation tanks, check dams, and farm ponds as 36, 39, and 21, respectively. Since agricultural land comprises up the majority of the study area, this research will help to enhance the irrigation system and increase the region's agricultural production. This study demonstrates that the integration of GIS can provide an efficient and effective platform to planners and decision makers for proper groundwater management and planning through convergent analysis of various data sets. 

## Data Availability

The authors confirm that the data supporting the findings of this study are available within the article and its supplementary materials. The data used in this research are available by the corresponding author upon reasonable request.
